# Mechanism of increased efficacy of recombinant Fc‐μTP‐L309C compared to IVIg to ameliorate mouse immune thrombocytopenia

**DOI:** 10.1002/jha2.304

**Published:** 2021-09-29

**Authors:** Bonnie J.B. Lewis, Beth Binnington, Megan Blacquiere, Rolf Spirig, Fabian Käsermann, Donald R. Branch

**Affiliations:** ^1^ Donald R. Branch, BS, MT(ASCP)SBB, PhD Donald R. Branch, BS, MT(ASCP)SBB, PhD 30 Bond Street, Keenan Research Centre Toronto M5B 1W8 Canada; ^2^ Department of Laboratory Medicine and Pathobiology University of Toronto Toronto Canada; ^3^ Research, CSL Biologics Research Center CSL Behring AG Wankdorfstrasse 10 Bern 3010 Switzerland; ^4^ Department of Medicine University of Toronto Toronto Canada

**Keywords:** immune thrombocytopenia, immunotherapy, ITP, ITP intravenous immunoglobulin, IVIG, IVIg, recombinant Fc hexamer

## Abstract

Recombinant Fc‐μTP‐L309C is more efficacious than intravenous immunoglobulin (IVIg) at ameliorating antibody‐mediated autoimmune diseases through its effects on Fcγ receptors (FcγRs). Fc‐μTP‐L309C inhibited in‐vitro FcγR‐mediated phagocytosis 10^4^/10^5^‐fold better than IVIg. Fc‐μTP‐L309C, given subcutaneously, recovered platelet counts in an immune thrombocytopenia (ITP) mouse model to a higher degree than IVIg at a 10‐fold lower dose. We show, using confocal microscopy, that Fc‐μTP‐L309C binds to monocyte‐macrophages and is rapidly internalized, whereas, IVIg remains on the cell surface. Western blotting showed that internalized FcγRIII is degraded through a lysosomal pathway, and this reduction of cell surface FcγRIII is likely responsible for the increased efficacy to ameliorate ITP.

## 1 INTRODUCTION

Intravenous immunoglobulin (IVIg) or subcutaneous immunoglobulin (SCIg) are major replacement therapies for primary immunodeficiency [1] and, at doses 1‐2 g/kg, have immunomodulatory effects; IVIg is a first line treatment for autoimmune diseases such as immune thrombocytopenia (ITP) [2]. Although both F(ab’)_2_‐ and Fc‐dependent mechanisms have been suggested to be involved in the immunomodulatory effects of this therapy, research in the field has emphasized that the IgG Fc fragment is crucial for its anti‐inflammatory properties [3]. IVIg is pooled from the blood of thousands of human donors and manufactured via chromatographic processes to formulate a highly purified, polyclonal IgG product that is suitable for i.v. or s.c. applications [4]. Its manufacture requires highly specialized production facilities with a focus on pathogen safety. Moreover, its supply is dependent on the availability and the collection of human plasma, and it is subject to some natural variability. These challenges associated with growing product demand, production, and availability have provided incentives to develop various Fc constructs as potential alternatives to IVIg/SCIg for diseases where its mechanism has been suggested to be Fc‐dependent [3, 5, 6, 7], with Fc‐blockade, at least in part, responsible for its amelioration of ITP. Thus, various investigators have proposed using recombinant (r) Fc multimers as a therapy to replace the use of IVIg. One such multimer is rFc hexamer (termed Fc‐μTP‐L30C) which has been previously described and shown to have 10‐ to 20‐fold increased efficacy compared to IVIg in amelioration of ITP and rheumatoid arthritis in mouse models [3, 8].

In the work reported herein, we have shown that Fc‐μTP‐L309C can effectively block in vitro FcγR‐mediated phagocytosis >12,000‐fold better than IVIg using a mouse macrophage cell line and >112,000‐fold better using mouse peripheral blood‐derived monocytes. We show that the hexamer demonstrates similar efficacy when administered subcutaneously as well as intraperitoneally without any stress reaction, and we support a previous report [9] that the mechanism of Fc‐μTP‐L309C treatment in a mouse model of ITP that results in 10‐ to 20‐fold more efficacy than IVIg is a result of increased binding of Fc‐μTP‐L309C to FcγRIII causing internalization and degradation of the receptor via choroquine‐sensitive lysosomes.

## 2 RESULTS AND DISCUSSION

(see Supplementary data for Materials and Methods)

### 2.1 Fc‐μTP‐L309C is a better inhibitor of FcγR‐mediated phagocytosis in comparison to IVIg

**FIGURE 1:**
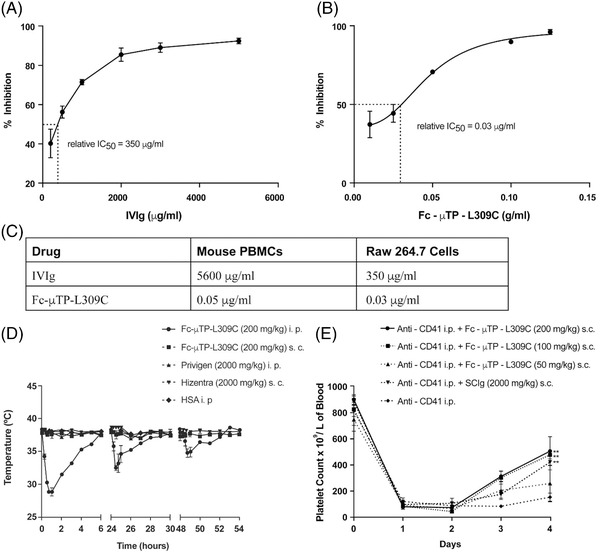
IC_50_s of IVIg and Fc‐μTP‐L309C to inhibit FcγR‐mediated phagocytosis. IVIg and Fc‐μTP‐L309C were used in the MMA to inhibit phagocytosis of sheep red blood cells (SRBC) opsonized with anti‐SRBC using mouse monocytes (from peripheral blood mononuclear cells (PBMCs) or the mouse macrophage cell line, RAW 264.7. **(A)** The IC_50_ curve of IVIg is shown to have an absolute IC_50_ of 350 μg/ml. Triplicate data are expressed as mean ± standard deviation (SD) as error bars. (**B)** The IC_50_ curve of Fc‐μTP‐L309C is shown to have an absolute IC_50_ of 0.03 μg/ml. Triplicate data were expressed as mean ± SD. (**C)** This table summarizes the data in panels (A) and (B), and compares the IC_50_ in mouse monocytes of IVIg, (5600 μg/ml), to Fc‐μTP‐L309C (0.05 μg/ml). (**D)** Best in vivo administration route of Fc‐μTP‐L309C given i.p. or s.c, compared with 2 g/kg of IVIg (i.p.) or SCIg (s.c.) at times 0, 24, and 48 h. Body (rectal) temperature was measured at 0, 15, 30, 45, and 60 min and at 2, 3, 4, 5, and 6 h after each injection with a thermometer. Shown are the average body temperatures; error bar indicates the range of temperatures (mean ± SD; *n* = 3 per treatment group). This experiment was repeated three independent times. (**E)** Treatment with a single s.c. dose of Fc‐μTP‐L309C (200 mg/kg, 100 mg/kg, or 50 mg/kg) or SCIg (2500 mg/kg) on day 2 in C57BL/6J mice with ITP. Shown are mean platelet (PLT) values in the blood; error bars indicate the mean ± SD; *n* = 6 for each treatment group. **indicates a *p* < 0.01. Similar results were obtained in two independent experiments

We have previously shown that a recombinant Fc hexamer, Fc‐μTP‐L309C, is 10‐fold more effective than IVIg to ameliorate platelet destruction in a mouse model of ITP and also to ameliorate inflammation in mouse models of arthritis [3, 8]. To begin to understand why the recombinant hexamer is so much better than IVIg, we first examined the efficacy of Fc‐μTP‐L309C to inhibit in vitro phagocytosis of antibody‐opsonized cells by mouse monocyte‐macrophages. To compare the abilities of IVIg and Fc‐μTP‐L309C to inhibit FcγR‐mediated phagocytosis, we used the monocyte monolayer assay (MMA) [10] to generate an average phagocytoic index (PI) for each at varying concentrations. We took the inverse of the average PI to generate a % inhibition at each concentration to generate a dose‐response. The concentration of each molecule at which 50% inhibition was observed (IC_50_ ) was determined using curve‐fitting software (GraphPad Prism). The IC_50_ of IVIg was 350 μg/ml (Figure 1A), whereas the IC_50_ of Fc‐μTP‐L309C was 0.03 μg/ml (Figure 1B) using the RAW264.7 mouse macrophage cell line. The IC_50_ of IVIg was 5600 μg/ml (Figure 1C), whereas the IC_50_ of Fc‐μTP‐L309C was 0.05 μg/ml (Figure 1C) using primary mouse monocytes.

These results appear to indicate increased binding avidity of Fc‐μTP‐L309C compared to IVIg. This supports our previous studies showing that the hexamerization of IgG1‐Fc lead to a dramatic increase in its binding avidity to FcγRs and inhibition of phagocytosis by THP1 cells, a monocyte cell line [3]. This finding is not unique to Fc‐μTP‐L309C, as it is also exhibited by other Fc multimers such as GL‐2045 [5], HexaGardTM [11], and recombinant trivalent human IgG1 Fc multimer (Fc3Y) [12]. However, our studies are the first to show using mouse monocyte‐macrophages such a dramatic difference in efficacy to inhibit the phagocytosis of opsonized RBCs.

### 2.2 Intraperitoneal injection of Fc‐μTP‐L309C but not subcutaneous delivery triggers body temperature decreases in mice

We next tested the efficacy of Fc‐μTP‐L309C compared to IVIg to ameliorate ITP in a mouse model. First, because we wanted to avoid any stress‐related reactions in our mice, we examined whether Fc‐μTP‐L309C induced any temperature drop in mice after different routes of administration (Figure 1D). Fc‐μTP‐L309C, given at 200 mg/kg via intraperitoneal (i.p.) injection triggered a rapid drop in the body temperature, which was fully recovered by 6 h (Figure 1D). However, we observed that in each consecutive administration, the temperature drop induced by i.p. injection of Fc‐μTP‐L309C was less pronounced (this experiment was repeated three consecutive times). A similar decrease in body temperature was absent when mice were treated with 200 mg/kg of Fc‐μTP‐L309C given subcutaneous (s.c.) Treatment with 2 g/kg of IVIg given i.p. and treatment with 2 g/kg of subcuntaneous immunoglobulin (SCIg; Hizentra, CSL Behring) given s.c. did not induce a temperature drop (Figure 1D).

Previous studies have shown that the hexamerization of Fcs to be associated with unwanted negative effects such as nonspecific FcγR signaling leading to intracellular activation [9, 12]. Ortiz et al. [12] showed that Fc multimers with two or three Fcs bound multiple FcγRs with high avidity but did not elicit FcγR signaling or calcium flux in monocytes. However, Fc multimers with five or more domains triggered dose‐dependent FcγR‐mediated signaling and calcium flux as well as rapid internalization of the Fc multimer via FcγRII [12]. Although Fc‐μTP‐L309C induced a temperature drop in mice when it was injected intraperitoneally, it did not induce a temperature drop when it was injected subcutaneously. Thus, it appears s.c. administration circumvented the potential stress‐related event that was observed with i.p. injection; thus, the s.c. route of injection became our preferred route of administration of Fc‐μTP‐L309C used in our ITP studies. Our results suggest that multimers having five or more Fc domains would not result in any adverse events if given s.c.

### 2.3 Fc‐μTP‐L309C provides therapeutic benefit in a mouse model of ITP when given subcutaneously

Using s.c. administration of Fc‐μTP‐L309C to mice given ITP via passive antibody administration of anti‐platelet antibody [13], we compared the therapeutic efficacy of Fc‐μTP‐L309C to SCIg. Fc‐μTP‐L309C was more effective at raising platelet counts in mice with ITP in comparison to SCIg at a 10‐fold lower dose (Figure 1E). Mice were treated with a single dose of Fc‐μTP‐L309C (200 mg/kg) given subcutaneously when the platelet nadir was attained 2 days after initiating antibody‐mediated depletion [13]. Treatment with Fc‐μTP‐L309C resulted in significantly increased platelet numbers, which were higher at days 3 and 4 than for mice treated with a 10‐fold larger dose of SCIg (2000 mg/kg) (Figure 1E).

### 2.4 Internalization and degradation of Fcγ‐receptors following Fc‐μTP‐L309C engagement

**FIGURE 2:**
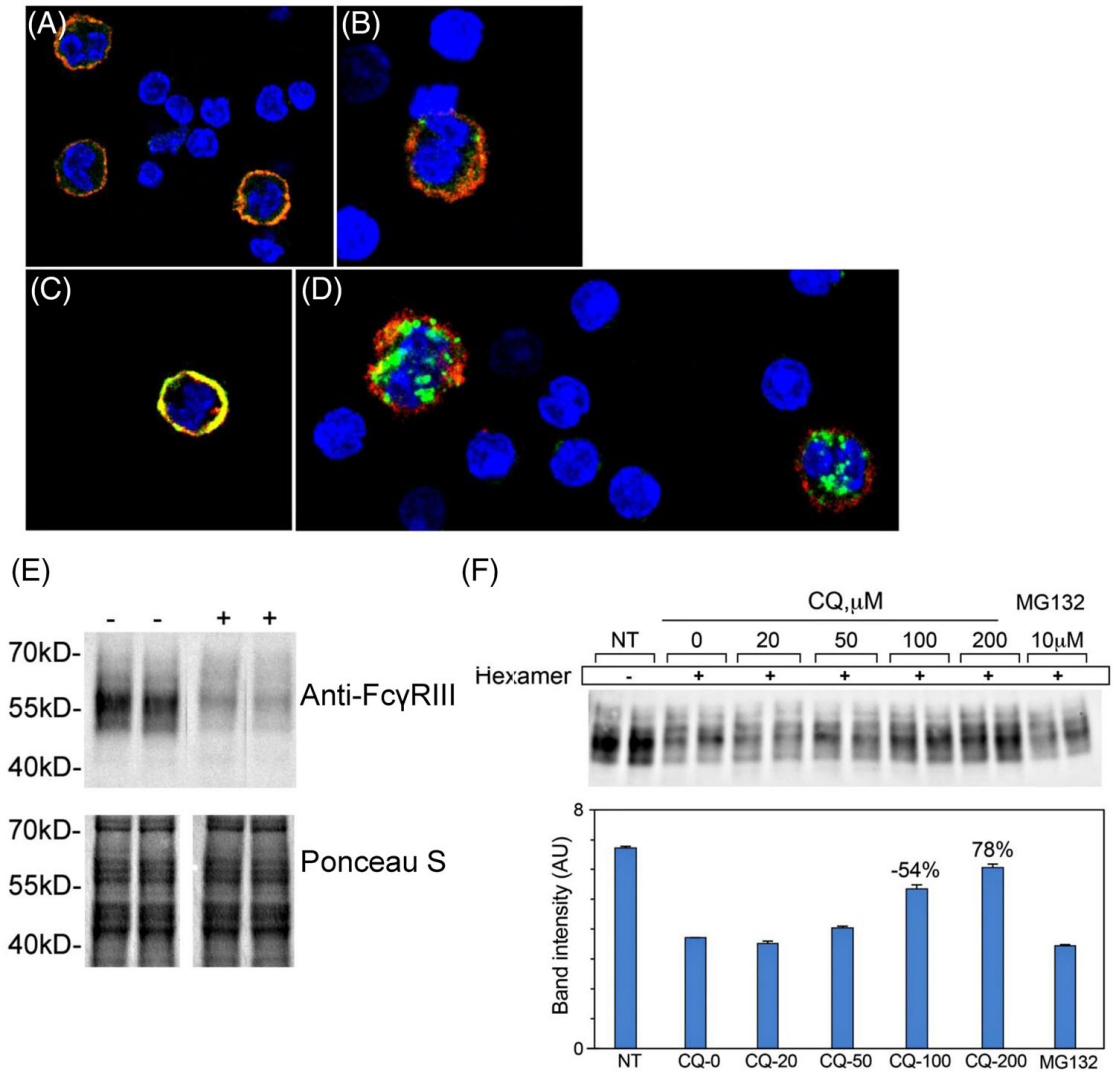
Internalization of Fc‐μTP‐L309C and subsequent degradation of activating FcγRIII. Monocytes from peripheral blood mononuclear cells (PBMCs) were incubated with green fluorescing AF488‐conjugated IVIg (**A and B**) or Fc‐μTP‐L309C (**C and D**), at either 4°C (**A and C**) or 37°C (**B and D**) for 30 min, followed by incubation with AF555‐conjugated F4/80 (red) to specifically label the monocytes, and 4',6‐diamidino‐2‐pheylindole (DAPI) to label cell nuclei (blue). Cells were then fixed and imaged by confocal microscopy. Representative images taken from one of three independent experiments is shown. (**E)** RAW 264.7 macrophages were incubated with 0 or 10 μg/ml Fc‐μTP‐L309C at 37°C for 3 h. Cell lysates (non‐reduced) were then analyzed by Western blotting using antibody against FcγRIII (CD16). Equal loading is shown by Ponceau S staining. One representative blot from three independent experiments is shown. (**F**) RAW 264.7 cells were pre‐treated in duplicate for 30 min with chloroquine diphosphate (CQ) or proteosome inhibitior, MG132, at the indicated concentrations prior to addition of 0 or 10 μM hexamer. Cells were incubated for 3 h at 37°C to allow hexamer binding and internalization. Cell lysates were analyzed by Western blotting as in (E). Band densities were quantified using National Institutes of Health (NIH Image J (bar graph). Error bars represent the range of duplicate values divided by 2. Percent protection from hexamer‐induced CD16 degradation observed with 100 μM or 200 μM CQ treatments is shown above the bar. CD16 band intensity observed in untreated cells and hexamer‐treated cells was defined as 100% and 0% protection, respectively

To understand why Fc‐μTP‐L309C was more efficacious than IVIg to inhibit in vitro FcγR‐mediated phagocytosis and had enhanced efficacy to ameliorate ITP, we examined what happens to these two molecules when they interact with the FcγR. A previous report showed that a similar recombinant Fc hexamer was able to bind to FcγRs and become internalized and degraded [11]. Therefore, to see if we could corroborate these earlier findings, we incubated mouse peripheral blood mononuclear cells with fluorescently labeled Fc‐μTP‐L309C or IVIg either at 4°C or 37°C for 30 min. The cells incubated with IVIg at 4°C (Figure 2A) or at 37°C (Figure 2B) showed a distinct halo indicating co‐localization of IVIg with cell surface F4/80 on monocytes but without internalization. However, with Fc‐μTP‐L309C, although a distinct plasma membrane co‐localization of Fc‐μTP‐L309C with F4/80 on monocytes was evident at 4°C (Figure 2C), considerable intracellular vesicles containing Fc‐μTP‐L309C as well as some surface Fc‐μTP‐L309C were evident at 37°C (Figure 2D). To test the fate of the FcγRs in this system, we performed Western blotting for FcγRIII after exposure to Fc‐μTP‐L309C for 3 h at 37 °C. Time 0 was used as a control as degradation induced by Fc‐μTP‐L309C was apparent after 1 h (data not shown). We observed significant degradation of the activating, FcγRIII after exposure to Fc‐μTP‐L309C (Figure 2E). We further show that the degradation of the Fc receptor is via the lysosomal and not proteosomal (ubiquitin) route since chloroquine blocked the majority of Fc‐μTP‐L309C‐induced FcγRIII degradation. (Figure 2F).

In conclusion, we have shown that Fc‐μTP‐L309C is superior to IVIg for 1. inhibiting FcγR‐mediated phagocytosis and 2. amelioration of ITP in a mouse model. Furthermore, we show that Fc‐μTP‐L309C administered s.c. does not induce a stress response and maintains its efficacy over IVIg. Finally, we show that the mechanism of enhanced amelioration of ITP is likely due to the increased binding avidity of the Fc‐μTP‐L309C to FcγRs compared to IVIg that results in the down‐regulation of the FcγR and its internal destruction by the monocyte‐macrophages which allows for increased circulation of antibody‐sensitized platelets. Our results raise the question as to how IVIg can effectively ameliorate ITP if it is so inferior at blocking FcγRs as our in vitro results indicate. It strongly suggests that FcγR‐blockade is not a major mechanism of IVIg efficacy for amelioration of ITP in the mouse model, that other mechanism(s) such as induction of immunomodulatory cytokines, such as interleukin‐11 [14], may be more important.

## Supporting information

Supporting Information
