# Supplementary material for: Mechanism of increased efficacy of recombinant Fc‐μTP‐L309C compared to IVIg to ameliorate mouse immune thrombocytopenia
Source: EJHaem. 2021 Sep 29;2(4):789–93. doi: 10.1002/jha2.304 (PMC9175896; doi:10.1002/jha2.304)
Supplement: Supplementary file 1 — Supporting Information [file JHA2-2-789-s001.docx]

**Supplementary data**

**Materials and Methods**

**Mice**

C57BL/6 (F, 6 weeks old) and BALB/c (F, 6 weeks old) mice were purchased from The Jackson Laboratory (Bar Harbor, ME). Mice were kept under a natural light-dark cycle, maintained at 22 ± 4°C, and fed with standard diet and water ad libitum. All experiments were performed after animal use protocols (AUP 1788) were approved by the University Health Network Animal Research Committee in Toronto.

**Biological Reagents**

The rat monoclonal anti-mouse GPIIb (CD41); clone MwReg30, rat IgG1λ) Ab used to induce ITP was purchased from BD PharMingen (Mississauga, ON). Privigen 10% IVIg, Hizentra 20% SCIg and Fc-µTP-L309C were from CSL Behring AG (Bern, Switzerland). Both Privigen and Hizentra are IVIg products, the former is normally used for intravenous injections while the latter is normally used for subcutaneous injection. IVIg and SCIg are used respectively. Rabbit anti-sheep IgG used to opsonize sheep RBCs in the MMA was purchased from MP Biomedicals (Solon, OH). Sheep RBCs were purchased from Lampire Biologicals, Inc (Everett, PA).

**Cell Isolation and Culture**

Mouse blood was obtained in accordance with the University Health Network Animal Research Committee (Toronto, ON). All methods were carried out in accordance with relevant guidelines and regulations. Mouse blood was obtained via cardiac puncture and PBMCs were also isolated from peripheral blood by density gradient centrifugation. RAW264.7 (RAW) cells (American Type Culture Collection, Manassas, VA) were maintained in RPMI 1640 (Sigma-Aldrich, St. Louis, MO) 10% fetal bovine serum (FBS) (Sigma-Aldrich), 4 mM L-glutamine (Sigma-Aldrich), penicillin (100 U/ml) (Sigma-Aldrich), and streptomycin (100 U/ml) (Sigma-Aldrich) (complete RPMI).

**Monocyte monolayer assay (MMA)**

The MMA was used to monitor the ability of IVIg or Fc-µTP-L309C to block FcγR-mediated phagocytosis, as described previously with minor modifications (Branch et al., 1984; Tong et al., 2016). Briefly, RAW cells or mouse PBMCs were counted and seeded into an 8-well tissue culture glass chamber slide (Nunc Lab-Tek II Chamber slides, VWR International, Mississauga, ON), and incubated for 1 hour at 37° C, in a 5% atmosphere of CO_2_. Meanwhile sheep RBCs were opsonized with rabbit anti-sheep IgG, (1 hr with mixing every 15 min., 37° C, 5% CO_2_). After 1hr of RAW or mouse monocyte adherence, non-adherent cells were removed and the cells were incubated with either IVIg or Fc-µTP-L309C in complete RPMI media for 1 hr at 37^o^C, 5% CO_2_. Opsonized sheep RBCs (suspended in 5% FBS/RPMI 1640) were then added to the chamber slide wells, and incubated (1 hr, 37 °C, 5% CO_2_). After incubation, the slides were vigorously washed with PBS, fixed in 100% methanol, air-dried, then mounted in Elvanol (Tong et al., 2016). Phagocytic events were manually quantified using a phase contrast microscope (40X) (Leica DM1000 Leica Microsystems, Richmond Hill, ON), Approximately 300 monocytes were counted per sample, and the phagocytic index (PI) of the test (with IVIg or Fc-µTP-L309C) or control (without inhibitors) was obtained by dividing the number of phagocytosed sheep RBCs by the number of monocytes or RAW cells and multiplying by 100 as previously described (Branch et al., 1984; Tong et al., 2016). PI values for each condition were the mean of triplicate wells. Percent inhibition at each drug dose was calculated according to the formula 100*(PI_control –_ PI_test_ )/ PI_control_ (Branch et al., 1984; Tong et al., 2016).

**ITP Mouse Model**

A dose-escalation mouse model of passive anti-platelet Ab (anti CD41/GPIIb, clone MWReg30) induced ITP was used, as described previously (Katsman et al., 2010). Briefly, mice were injected i.p. with 68 µg/kg of MWReg30 on days 0 and 1, followed by increasing doses of an additional 34 µg/kg each subsequent day. It has been well established that under these conditions, platelet nadir is achieved on day 2 and maintained until day 5 (Katsman et al., 2010; Leontyev et al., 2012; Leontyev et al., 2014). Platelets in whole blood samples were quantified daily using a calibrated flow cytometer (FACSCalibur, BectonDickinson, Franklin Lakes, NJ) as previously described (Katsman et al., 2010). For treatment of ITP, SCIg or Fc-µTP-L309C were given s.c., 2 hours after platelet Ab administration on day 2 of the experiment. The dose for SCIg was 2 g/kg for BALB/c or 2.5 g/kg for C57BL/6 mice, as optimized previously (Leontyev et al., 2012; Leontyev et al., 2014). Fc-µTP-L309C was administered at 200 mg/kg, 100 mg/kg, or 50 mg/kg. Human serum albumin (HSA) was used as a protein control at the same doses as SCIg.

**Stress Response**

Body temperature was used to assess the occurrence of a stress response induced by different routes of administration of IVIg, SCIg and Fc-µTP-L309C. Briefly, mice were injected i.p. with 2 g/kg of IVIg or s.c. with 2 g/kg of SCIg. Additionally, mice were injected i.p. or s.c. with 200 mg/kg of Fc-µTP-L309C. Body (rectal) temperature was monitored at 0, 15, 30, 45, and 60 minutes and at 2, 3, 4, 5, and 6 hours post injection using a model TK-610B thermometer equipped with a thermocouple probe (Harvard Apparatus, USA). HSA was used as a protein control at the same doses as IVIg/SCIg.

**Statistical Analysis**

Statistical analyses were performed using GraphPad Prism (GraphPad Software, Version 6.0, San Diego, CA). Data are presented as mean ± standard deviation (SD) using student’s t-test.

**Effect of hexamer internalization on CD16 expression measured by western blot**

**Cell treatments**

RAW 264.7 cells were cultured in RPMI supplemented with 10% heat inactivated fetal calf serum, 10mM HEPES, antibiotics (100 IU penicillin/100 µg/mL streptomycin), 2mM Gluta-Plus and grown at 37^o^C, 5% CO_2_ in a humidified incubator. All cell culture media and additives were purchased from Wisent Bioproducts, St. Bruno QC.

Cells were grown overnight in 24-well plates until about 80-90% confluent. The next day, fresh media was added containing 0-200 µM chloroquine (CQ) disphosphate, or 10 µM MG132 (both from Sigma-Aldrich, Oakville, ON), diluted from 10 mM stock solutions in water or DMSO, respectively (final DMSO concentration 0.1%). Cells were incubated with drugs at 37^o^C for 30 min then hexamer was added to a final concentration of 10µg/mL (diluted from 1 mg/mL stock in water).

Control wells with no hexamer, drugs only, or 0.1% DMSO were included. Cells were returned to the incubator and hexamer was allowed to bind/internalize continuously for 3hr. The cell plate was then chilled on ice, media was aspirated, and cells were rinsed 3x in ice-cold PBS. Washed cells were lysed 30 min on ice in 0.1mL RIPA containing protease inhibitor cocktail (2mM AEBSF, 130 µM bestatin, 14 µM E-64, 1 µM leupeptin, 0.3 µM aprotinin (Bioshop, Burlington, ON)).

Lysates were collected, spun at 10,000xg 10 min at 4 ^o^C, and protein in the soluble fraction was measured using the Pierce BCA reagent (Fisher Scientific, Ottawa, ON).

**Western blotting**

Trial western blots were performed on reduced or non-reduced RAW 264.7 lysates using mAb rabbit anti-mouse CD16/FcꙋR3 clone R002 (Sino Biological, Wayne PA). Clone R002 did not bind well to reduced CD16, but gave a pronounced band at ~50kD in non-reduced samples which is consistent with the expected size. Though the predicted mass of CD16 is about 30kD, the protein is known to be glycosylated. Human CD16 detected at 50kD migrates at about 30kD after deglycosylation (Patel, 2018). Subsequent experiments were performed using non-reduced samples.

For SDS-PAGE, 15 µg protein samples were separated on a 10% acrylamide gel then transferred 1hr at 100 V to PDVF. Membranes were stained with Ponceau S to verify equal loading/transfer, then destained and blocked with 2% BSA in 20mM Tris-buffered saline containing 0.5% NP-40. CD16/FcꙋRIII was detected with 0.5 µg/mL mAb clone R002 followed by goat anti-rabbit-HRP diluted 1/10,000 (Bio-Rad, Mississauga ON). ECL detection of antibody binding was performed with Bio-Rad Clarity ECL blotting substrate and blots were imaged using a Bio-Rad Chemidoc imager.

**Analysis of Fc-µTP-L309C Internalization**

Mouse PBMCs were isolated from peripheral blood (obtained by cardiac puncture) by density gradient centrifugation using Ficoll. Fc-µTP-L309C and IVIg were fluorescently tagged using an Ab labelling kit (Thermo Fisher Scientific, Alexa Fluor^®^ 488 Ab Labeling Kit) according to the manufacturer’s instructions. PBMCs were immobilized onto glass microscope slides using a Cytospin, Washed,then incubated 30 min. at either 4 °C (surface binding) or 37 °C (to allow for internalization to occur) with either Alexa488-Fc-µTP-L309C or Alexa488-IVIg. PBMC’s were then washed and fixed with 4% paraformaldehyde (PFA) in PBS for 10 minutes, before further labelling. Monocytes were labelled with a rat anti-mouse F4/80 Ab (clone A3-1, Bio-Rad, Hercules, CA), followed by a goat anti-rat IgG (H+L) antibody conjugated to Alexa Fluor^®^ 555 (Thermo Fisher Scientific). Nuclei were labelled with 300nM DAPI in PBS (Thermo Fisher Scientific) then slides were mounted using Prolong Diamond anti-fade mountant.. Images were acquired using a Leica SP8 Confocal microscope with a 63X objective (Hospital for Sick Children Imaging Facility, Toronto, ON). Laser power and acquisition settings were kept constant between image acquisitions of different conditions. Images shown are representative of cells from three independent experiments.
